# Successful treatment of radiotherapy and apatinib in patient with mediastinal mixed non-seminomatous germ cell tumor: A case report

**DOI:** 10.1097/MD.0000000000027617

**Published:** 2021-10-29

**Authors:** Congcong Ren, Jing Zhao, Lin Kang, Yan Di, Gang Qiu, Qingxue Wang

**Affiliations:** a Department of Oncology, Hebei General Hospital, Shijiazhuang, Hebei, China; b Graduate School, Hebei North University, Zhangjiakou, Hebei, China; c Department of Pathology, Hebei General Hospital, Shijiazhuang, Hebei, China.

**Keywords:** apatinib, mediastinal non-seminomatous germ cell tumor, radiotherapy, targeted therapy, volumetric-modulated arc therapy

## Abstract

**Rationale:**

Mediastinal non-seminomatous germ cell tumors (MNSGCTs) are rare malignancies. Chemotherapy followed by surgical resection has been regarded as the standard management, but treatment options for chemotherapy-refractory patients or those with unresectable tumors are limited, resulting in a very poor prognosis.

**Patient concerns:**

An 18-year-old female presented with symptoms of cough, chest tightness, and shortness of breath for 2 months, and the symptoms gradually worsened.

**Diagnosis:**

Computed tomography (CT) revealed a large mediastinal mass invading the pericardium and great blood vessels. Serum human chorionic gonadotropin (HCG) and α-fetoprotein (AFP) levels were normal. Histopathological examination of biopsy specimens revealed mixed MNSGCT with embryonal carcinoma and immature teratoma components.

**Interventions:**

The patient achieved complete remission (CR) and long-term survival after multimodal therapy comprising chemotherapy, positron emission tomography/CT (PET/CT)-guided volumetric-modulated arc therapy (VMAT), and anti-angiogenic targeted therapy.

**Outcomes:**

The patient was followed up for more than 4 years without recurrence, metastasis, or treatment-related adverse effects.

**Lessons:**

The case presented here highlights the importance of multidisciplinary diagnosis and treatment, providing evidence that radiotherapy and anti-angiogenic therapy may play an important role in unresectable or residual tumors after failure of conventional treatments of MNSGCT. Percutaneous biopsy is necessary for diagnosis if the tumor is unresectable, and serum AFP and HCG levels are normal. Additionally, PET/CT is an effective method for evaluation of efficacy and radiotherapy guidance for patients with MNSGCTs.

## 1 Introduction

Extragonadal germ cell tumors (GCTs) are relatively rare and mostly occur in the mediastinum and retroperitoneum. Primary malignant mediastinal germ cell tumors (MGCTs) account for 1% to 3% of GCTs and have similar histological features, serum tumor markers, and characteristic genetic abnormalities as their gonadal counterparts.^[1–3]^ In the past several decades, little progress has been made in the treatment of GCTs. For mediastinal non-seminomatous GCTs (MNSGCTs), platinum-based chemotherapy followed by surgical resection of residual tumor has been regarded as the standard management at present, but the prognosis of MNSGCTs is inferior to that of the gonads or other extragonadal sites, with a 5-year survival rate ranging from 20% to 50%.^[1,2]^ However, treatment options for chemotherapy-refractory patients or those with unresectable tumors are limited, which leads to a worse prognosis. Because of the rarity of primary MNSGCT, there have been no large-scale studies comparing different treatment strategies, and no other novel systemic therapies have been recommended, except for chemotherapy.^[3]^

Here, we report a chemotherapy-refractory and unresectable MNSGCT patient who was cured using multimodal treatment comprising chemotherapy, positron emission tomography/CT (PET/CT)-guided volumetric-modulated arc therapy (VMAT), and anti-angiogenic targeted therapy. In addition, we reviewed the relevant literature and integrated the epidemiological characteristics, diagnosis, prognosis, and treatments, which would further advance knowledge and improve treatment strategies for MNSGCT.

## 2 Case report

### 2.1 Ethic and informed consent

This case report was approved by the institutional review board of the Hebei General Hospital. Informed consent was obtained from the patient and her parents for publication of this case report and accompanying images.

### 2.2 Case presentation

**Figure 1:**
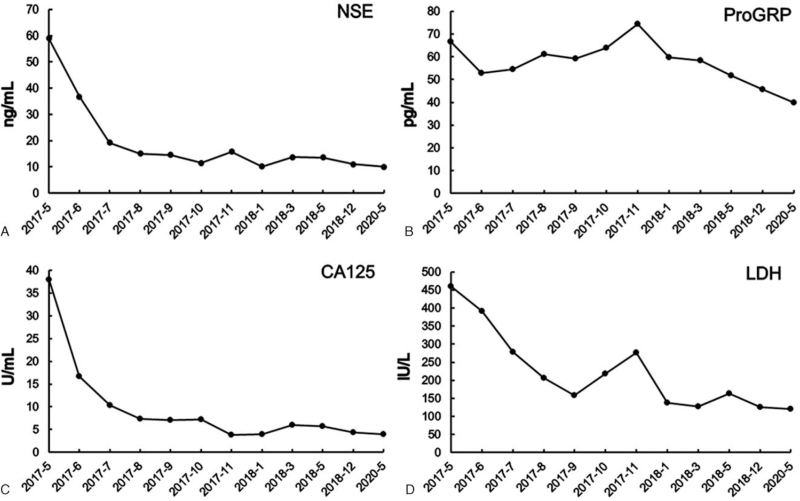
The levels of serum NSE (A), ProGRP (B), CA125(C) and LDH (D) during treatment. CA125 = carbohydrate antigen 125; LDH = lactate dehydrogenase; NSE = neuron-specific enolase; ProGRP = pro-gastrin-releasing peptide.

**Figure 2:**
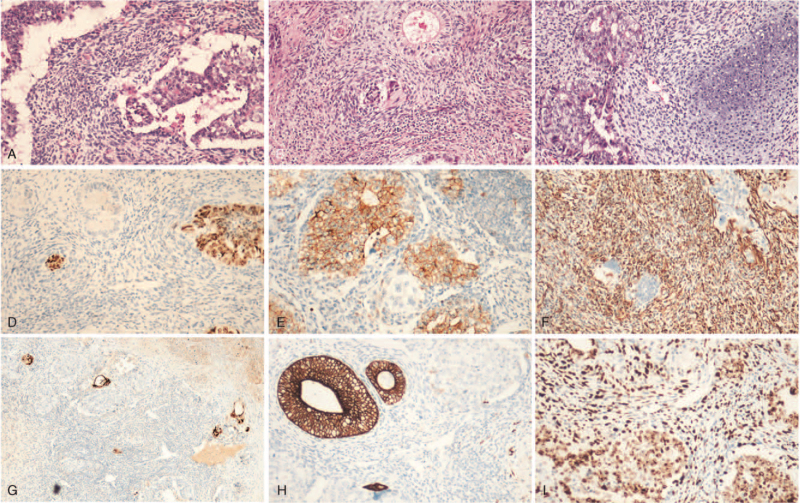
Histopathology and immunohistochemistry of the tumor tissue (magnification 100×). The H&E staining shows embryonal carcinoma containing pleomorphic cells growing in solid and glandular patterns (A), admixed with immature teratoma containing immature neuroectodermal tubules (B) and mature cartilage (C). Immunohistochemistry indicates OCT3/4 stains germ cell tumors in a nuclear pattern (D), CD30 stains embryonal carcinoma in a membranous pattern (E), vimentin stains the mesenchymal component of teratomas in cytoplasmic pattern (F), NSE (G) and Syn (H) stain primitive neural tubes in cytoplasmic pattern, and Ki-67 stains in a nuclear pattern (I).

**Figure 3:**
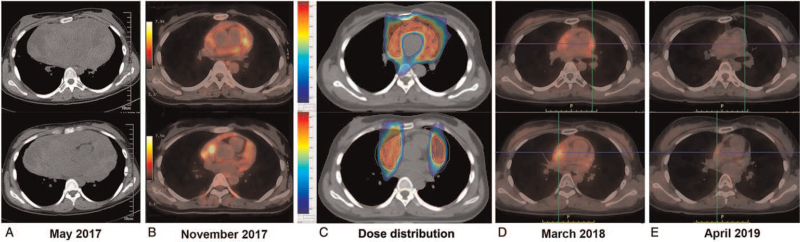
Imaging findings of the tumor during treatment and dose distribution of irradiation target volume. CT scan showed a huge mediastinal mass with invasion of pericardium and great blood vessels before treatment (A). PET/CT revealed the tumor significantly shrank and the SUVmax was 12.6 (B). Colorwash of dose distribution in radiotherapy plan. The red area represents 95% of the PTV receiving a prescription dose of 50 Gy (C). PET/CT indicated small amount of viable tumor tissues existed, SUVmax significantly reduced (SUVmax 1.8–3.9) after radiotherapy (D). PET/CT showed the mediastinal residual mass almost completely diminished and SUVmax was further reduced (SUVmax 1.4) 6 months after the end of apatinib targeted therapy (E).

An 18-year-old female presented with a two-month history of cough, chest tightness, and shortness of breath, and was hospitalized on May 8, 2017, as the symptoms worsened. On physical examination, her Eastern Cooperative Oncology Group (ECOG) performance status was 2, body temperature was 36.4°C, pulse was 110 beats/min, respiratory rate was 24 breaths/min, and blood pressure was 83/66 mm Hg. There was no peripheral lymphadenopathy. The breath sounds were bilaterally diminished; no added sounds were heard, and the heart sounds were distant. Abdominal examination revealed no abnormalities. Laboratory test results revealed elevated levels of serum neuron-specific enolase (NSE) (58.94 ng/mL, normal range: 0–15 ng/mL), pro-gastrin-releasing peptide (ProGRP) (63.55 pg/mL, normal range: <50 pg/mL), carbohydrate antigen 125 (CA125) (37.99 U/mL, normal range: <35 U/mL), and lactate dehydrogenase (LDH) (391.7 IU/L, normal range: 120–250 IU/L) (Fig. 1). Other cancer biomarkers, including α-fetoprotein (AFP) and human chorionic gonadotropin (HCG), were within normal limits. Her gynecological ultrasound findings were normal. Echocardiography indicated a pericardial solid mass and a small amount of pericardial effusion, decreased left ventricular diastolic function, left ventricular ejection fraction of 54%, and pulmonary artery systolic pressure (PASP) of 42 mm Hg. Computed tomography (CT) revealed a huge mass measuring 10 cm × 8.9 cm × 11 cm in the mediastinum with invasion of surrounding structures, including the pericardium and great blood vessels; multiple enlarged lymph nodes in the mediastinum; thickened pericardium with a small amount of pericardial effusion; and no distant disease (Fig. 3A). The bloody pericardial effusion was drained, and a CT-guided percutaneous needle biopsy was performed. Hematoxylin-eosin (H&E) staining of tumor tissue sections revealed multiple malignant primordial germ cell components, which were composed of embryonal carcinoma containing pleomorphic cells growing in solid and glandular patterns, and immature teratoma containing immature neuroectodermal tissue and mature cartilage (Fig. 2A–C). Immunohistochemistry confirmed OCT3/4 positive in germ cell tumors; CD30 positive in embryonal carcinoma; vimentin positive in the mesenchymal component of teratomas; NSE and Syn positive in the primitive neural tube; Ki-67 highly expressed in all these components; and HCG and AFP negative (Fig. 2D–I). The final diagnosis was primary malignant mixed MNSGCT, of which ∼60% were embryonal carcinoma and 40% were immature teratoma.

The patient received 6 cycles of chemotherapy with EP regimen (etoposide 100 mg/m^2^ and cisplatin 20 mg/m^2^ daily for 5 days, every 21 days). The symptoms of cough and dyspnea resolved, and the tumor markers LDH, NSE, and CA125 gradually normalized, but ProGRP levels still remained higher than normal (Fig. 1). Chest CT showed that the soft tissue mass in the mediastinum was significantly reduced. Positron emission tomography/CT (PET/CT) performed one month after the end of chemotherapy revealed an uneven highly metabolic soft tissue mass around the great vessels of the mediastinum and in the pericardium, in which multiple areas of cystic necrosis and calcification were seen, which was significantly shrunken compared to that prior to chemotherapy (Fig. 3B). The maximum standardized uptake value (SUVmax) of the mass was 12.6, considering that the active tumor tissue remained. Efficacy was evaluated as partial response (PR) according to the response evaluation criteria in solid tumors vision 1.1 (RECIST 1.1). However, the surgical oncology team assessed that there was no indication for radical resection because of extensive pericardium and great blood vessel involvement. Therefore, PET/CT-guided VMAT was administered with a linear accelerator using 6-MV photons to deliver a total dose of 50 Gy in 25 fractions over five weeks from November 9, 2017, to December 13, 2017. Involved-site radiation therapy (ISRT) was used to delineate the target area, described as follows: the gross tumor volume (GTV) was the residual tumor visible on PET/CT images after chemotherapy, the clinical target volume (CTV) included the GTV and the invaded tissues reference to pre-treatment CT scan, and the CTV was expanded 0.5 cm to generate a planning target volume (PTV). The dose volume histogram (DVH) revealed that 95% of the PTV received a prescription dose of 50 Gy (Fig. 3C). All organs at risk (OARs) received doses within their tolerance, such as the percentage volume of the lung that received >20 Gy was <25% (V20 <25%) and the mean dose was <12 Gy; and the percentage volume of the heart that received >30 Gy was <40% (V30 < 40%) and the mean dose was <26 Gy. The patient successfully completed radiotherapy without any acute or long-term toxicities. A CT scan 1 month after radiotherapy showed that the mediastinal soft tissue mass was slightly reduced.

PET/CT was performed 3 months after radiotherapy on March 13, 2018, and indicated that there was still viable residual tumor tissue, but the tracer accumulation was decreased (SUVmax 1.8–3.9) (Fig. 3D). Echocardiography indicated a left ventricular ejection fraction of 73% and a PASP of 30 mm Hg. Efficacy was assessed to maintain PR after radiotherapy. Surgeons then reassessed that the mass could not be cured by surgery. After obtaining informed consent from the patient and her parents, we initiated anti-angiogenic therapy with oral apatinib 500 mg per day combined with oral etoposide capsule 100 mg per day for 10 days, every 21 days, on March 17, 2018. After three cycles of etoposide combined with apatinib, the regimen was changed to apatinib alone with a dose reduction to 250 mg per day due to side effects such as nausea, hoarseness, mild deranged liver function, grade 3 hypertension, grade 2 hand-foot skin reaction (HFSR), and grade 2 proteinuria (2.4 g/24 h, normal range: <150 mg/day). The low dose of apatinib (250 mg per day) lasted until October 2018 and was well tolerated; the patient has received no further interventions since then. PET/CT in April 2019 revealed that the mediastinal residual mass almost completely diminished and the metabolism was further reduced (SUXmax 1.4), considering that tumor activity was inhibited (Fig. 3E). Up until follow-up in June 2021, the patient maintained complete remission without evidence of recurrence and treatment-related adverse effects, and gave birth to a child.

## 3 Discussion

### 3.1 Epidemiological characteristics

Primary malignant MGCTs are relatively rare extragonadal GCTs, which usually occur in males aged 20 to 40, accounting for 1% to 4% of mediastinal tumors and 1% to 3% of GCTs.^[4]^ The etiology is unclear and it has been suggested that MGCTs are derived from primitive germ cells that migrate aberrantly along the urogenital ridge during the early stage of embryogenesis.^[1]^ Primary malignant MGCTs have similar histological features, serum tumor markers, characteristic genetic abnormalities, and prognosis as their gonadal counterparts. Histologically, MGCTs are divided into two categories: mediastinal seminomas (MSGCTs) and mediastinal non-seminomas (MNSGCTs). MNSGCTs include immature teratomas, yolk sac tumor, embryonal carcinoma, choriocarcinoma, and mixed germ cell tumors (two or more types of germ cell tumors coexist).^[4]^

### 3.2 Diagnosis and prognosis

Primary malignant MNSGCTs are insidious and may be asymptomatic in the early stage, whereas the majority of patients exhibit mediastinal compression symptoms such as cough, chest tightness, shortness of breath, and chest pain.^[5,6]^ Serum HCG and AFP are elevated in ∼30% to 35% and 60% to 80% of patients with extragonadal GCT, respectively.^[7]^ It has been suggested that MNSGCT can be clinically diagnosed without biopsy based on young men, large mediastinal masses, and significantly elevated serum tumor markers (HCG > 5000 U, AFP > 100 0 U).^[8]^ It was reported that CEA and CA19–9 levels increased in some patients, but these tumor markers lacked diagnostic clinical usefulness.^[7]^ Therefore, diagnostic biopsy is necessary if serum AFP and HCG levels are normal. In our case, CT-guided percutaneous biopsy of the mediastinal mass was performed because of the normal level of serum HCG and AFP, while serum LDH, CA125, NSE, and ProGRP were elevated initially. The histopathology suggested that the patient had a rare case of mixed malignant MNSGCT with high proliferative activity, composed of embryonal carcinoma and immature teratoma. Immunohistochemical staining proved that the tumor markers were produced by tumor cells; therefore, we speculated that the production of NSE, ProGRP, and CA125 may be as a result of the differentiation to neural tissue and glandular epithelial cells by the pluripotent cells of origin. According to the prognostic evaluation criteria of the International Germ Cell Cancer Collaborative Group (IGCCCG), the prognosis of this patient is poor, with a 5-year survival rate of 40%.^[9]^

### 3.3 Treatments

Platinum-based chemotherapy followed by surgical resection of residual tumors is currently regarded as the standard management for primary MNSGCTs.^[10]^ Before the introduction of cisplatin-based regimens, the median survival of patients with primary MNSGCT was 6 months.^[11]^ Although BEP (bleomycin/etoposide/cisplatin) is the predominant regimen used in NSGCT, some studies have established EP or VIP as standard regimens to prevent pulmonary complications.^[12–14]^ In our case, due to the young age and poor remaining lung function of the patient, we chose chemotherapy with EP regimen in order to reduce pulmonary toxicity. However, despite standard chemotherapy and residual tumor resection, patients with primary MNSGCT rarely achieved long-term disease-free survival, with long-term survival rates ranging from 40% to 50%.^[15]^ Furthermore, aggressive surgery for primary malignant MNSGCT may require a cardiopulmonary bypass or major mediastinal vessel replacement; thus, giant mediastinal tumors are difficult to remove and the experience of the surgical oncology team is a crucial factor affecting the prognosis.^[16,17]^ In contrast, considering the development of modern radiation technology, radiotherapy is relatively easy to implement, and VMAT technique can target all residual lesions, even those close to important mediastinal structures, and reduce the exposure dose to normal tissues. However, in clinical practice, radiotherapy is rarely used as a treatment method in NSGCT, and limited data are available regarding its effectiveness, optimal dose, fields, and targeted area. Wang J et al.^[1]^ retrospectively analyzed 61 patients with primary malignant MNSGCT, 22 of whom underwent radiotherapy (median dose, 52 Gy) in the initial treatment. The results revealed that patients who received radiotherapy showed improved 5-year overall survival (OS) (68.2% vs 38.5%, *P* = 0.043), progression-free survival (PFS) (45.5% vs 20.5%, *P* = 0.023), and local recurrence-free survival (LRFS) (77.3% vs 38.5%, *P* = 0.003) compared to those who did not. Additionally, they recommended a high dose of 55 to 60 Gy. Kitsukawa et al^[7]^ reported a case of mediastinal embryonic carcinoma with cerebellar metastases that disappeared completely after 30 Gy of radiotherapy. Kita et al^[18]^ reported complete clinical remission of a residual retrocrural NSGCT with 60 Gy delivered by intensity-modulated radiation therapy (IMRT). In our case, radical resection of the residual tumor was difficult because of extensive pericardium and great blood vessel involvement; thus, radiotherapy using the VMAT technique was administered to reduce the exposure dose to normal tissues. In addition, only a prescribed dose of 50 Gy was administered to avoid radiation-induced constrictive pericarditis. However, in this patient, PET/CT after radiotherapy indicated that there was still a viable residual tumor with significantly reduced activity. Our results indicate that radiotherapy is an effective local treatment option and undoubtedly the best choice for patients with MNSGCT who cannot undergo radical resection, and a prescribed dose of more than 50 Gy is recommended. Moreover, it is noteworthy that PET/CT was adopted in the present case to detect active residual tumor due to the poor sensitivity and specificity of serum tumor markers and CT. PET/CT seems to be useful for MNSGCT patients with negative tumor markers or patients evaluated as having stable disease (SD) and PR on CT after treatment.^[19]^

Angiogenesis is essential for tumor growth and metastasis, and vascular endothelial growth factor (VEGF) and its receptors (VEGFRs) play a crucial role.^[20]^ Of the three different tyrosine kinase receptors, VEGFR1; VEGFR2; and VEGFR3, VEGFR2 plays a pivotal role in VEGF-mediated cancer angiogenesis.^[21]^ When binding to VEGF, the dimerization of VEGFR2 causes autophosphorylation of intracellular tyrosine kinase domains, leading to activation of the PLC-g-Raf kinase-MEK-MAP kinase pathway, which promotes the proliferation of vascular endothelial cells to develop new blood vessels in tumor tissues, thereby ensuring the supply of oxygen and nutrients and leading to the growth and metastasis of cancer.^[21]^ In recent years, targeted therapy has been investigated in GCTs, but most of the results have been discouraging.^[22]^ The efficacy of anti-angiogenic agents, such as paczopanib,^[23]^ thalidomide,^[24]^ lenalidomide,^[25]^ and bevacizumab,^[22]^ was also limited, and objective response was rarely achieved. However, these studies only focused on the efficacy of anti-angiogenic agents used alone. Data on the use of anti-angiogenic agents in combination with other treatments have not been reported. Apatinib is a novel anti-angiogenic agent that specifically targets VEGFR2.^[26]^ This oral small-molecule tyrosine kinase inhibitor was approved as a second-line treatment for advanced gastric cancer in the People's Republic of China in 2014.^[27]^ It is currently used in the treatment of a variety of solid tumors, such as lung cancer, hepatocellular carcinoma, and breast cancer.^[27–29]^ Furthermore, it has been reported that apatinib can reverse P-glycoprotein (ABCB1)- and breast cancer resistance protein (ABCG2)-mediated multidrug resistance (MDR) by inhibiting its transport function, and may be useful in circumventing MDR to other conventional antineoplastic drugs.^[30,31]^ In our case, the giant mediastinal tumor was found to be rich in blood supply through contrast-enhanced CT and biopsy. Therefore, for the residual lesions after chemotherapy and radiotherapy, we initiated a combined regimen using apatinib anti-angiogenic therapy and etoposide capsule chemotherapy, based on the consideration that the expression of VEGFR receptor cannot be examined as only stained slides of tumor were available, and we also hoped to obtain a synergistic effect. The initial dose of apatinib was 500 mg per day, but it was reduced to 250 mg due to side effects of hoarseness, hypertension, HFSR, and proteinuria. The standard dose of apatinib in patients with gastric cancer is 850 mg, while the common application dose is 500 mg according to the use in of NSCLC and SCLC.^[32,33]^ This suggests that the tolerance of different tumor species to apatinib is different, hence, the application dose should be individualized and an initial dose of 250 mg can also be selected clinically. In this report, low-dose apatinib monotherapy eventually eliminated residual tumors in a patient with primary MNSGCT. Since many other anti-angiogenic agents are yet to show an ideal therapeutic effect in primary MNSGCT, the distinct effect of apatinib observed in this case may not be entirely attributable to VEGF/VEGFR-mediated angiogenesis.^[21]^ In addition, several studies^[34,35]^ have demonstrated that apatinib can reverse MDR in a variety of tumors. As for the present case, the high efficacy may be partly related to the combination of etoposide capsule and apatinib, which may reverse the resistance of etoposide. The mechanism of action of apatinib on MNSGCT is complicated and warrants further research.

## 4 Conclusions

Unresectable mixed MNSGCT is clinically rare and refractory, with a high recurrence rate and poor prognosis. Our data provide evidence that in order to reduce local recurrence and improve survival, radiotherapy may be an alternative to surgery, or a treatment option for residual tumors after chemotherapy and surgery. A prescribed dose of more than 50 Gy may be necessary, and PET/CT is an effective method for radiation guidance and efficacy evaluation in MNSGCT patients. Moreover, apatinib might be effective in patients with chemotherapy-refractory MNSGCT, and anti-angiogenesis in combination with others would be a promising therapy for MNSGCT; however, further studies are still needed. Additionally, percutaneous biopsy is necessary to diagnose tumor unresectability, and determine levels of serum AFP and HCG levels.

## Author contributions

**Conceptualization:** Jing Zhao.

**Data curation:** Congcong Ren, Jing Zhao, Yan Di, Qingxue Wang.

**Formal analysis:** Jing Zhao, Lin Kang, Gang Qiu.

**Investigation:** Congcong Ren, Jing Zhao.

**Methodology:** Congcong Ren, Jing Zhao, Lin Kang, Yan Di, Gang Qiu.

**Validation:** Jing Zhao, Gang Qiu.

**Visualization:** Congcong Ren, Jing Zhao, Lin Kang.

**Writing – original draft:** Congcong Ren, Jing Zhao, Yan Di.

**Writing – review & editing:** Congcong Ren, Jing Zhao.
